# Supplementary figures and images for: Evaluation of stress-controlled high-cycle fatigue characteristics in PLA-wood fused deposition modeling 3D-printed parts under bending loads
Source: PLoS One. 2024 Apr 18;19(4):e0300569. doi: 10.1371/journal.pone.0300569 (PMC11025761; doi:10.1371/journal.pone.0300569)

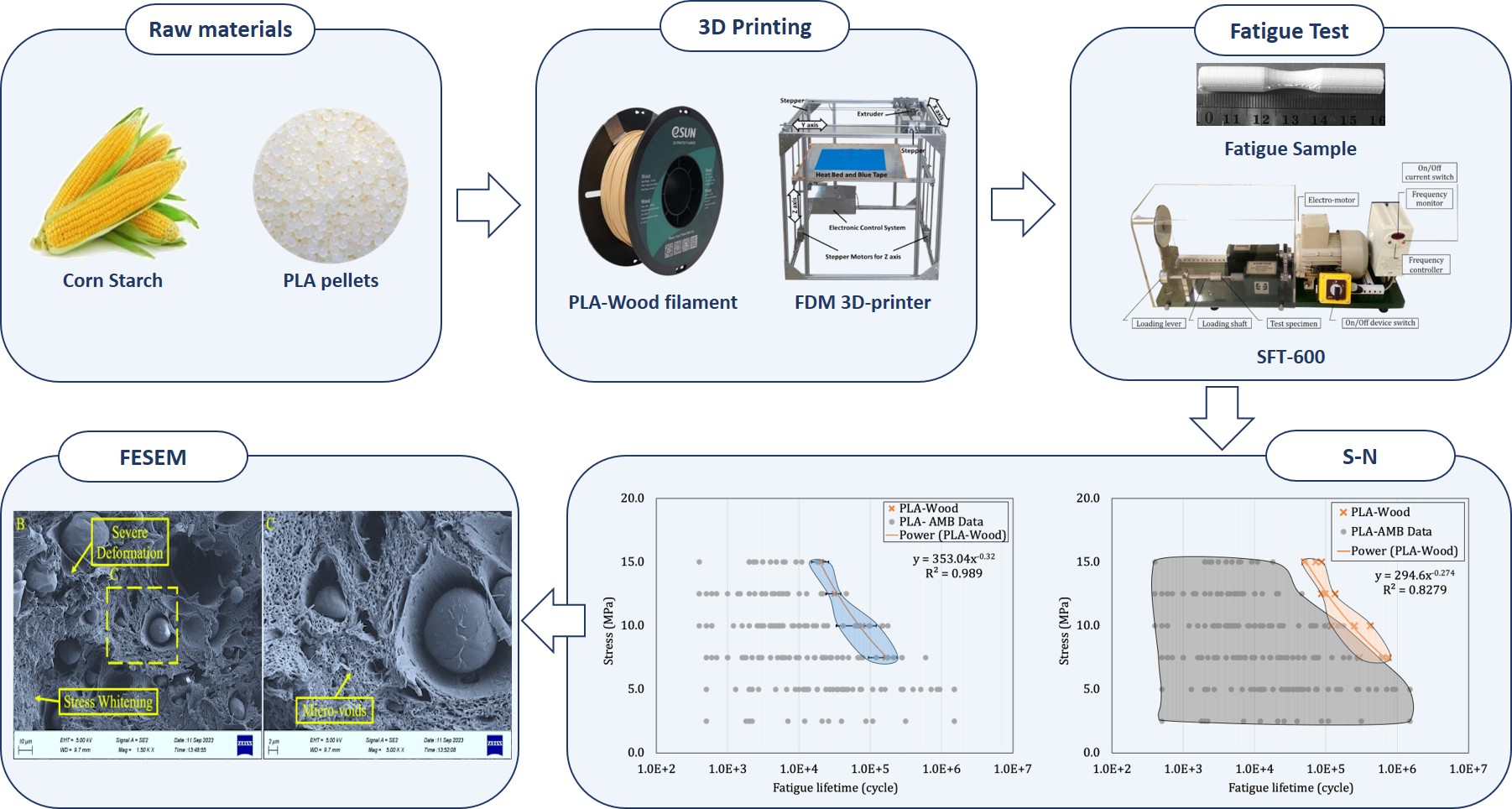

Supplement: S1 Graphical abstract — (JPG) [file pone.0300569.s001.jpg]
