# Supplementary material for: Evaluation of stress-controlled high-cycle fatigue characteristics in PLA-wood fused deposition modeling 3D-printed parts under bending loads
Source: PLoS One. 2024 Apr 18;19(4):e0300569. doi: 10.1371/journal.pone.0300569 (PMC11025761; doi:10.1371/journal.pone.0300569)
Supplement: Attachment — Submitted filename: Review comments - PONE.pdf [file pone.0300569.s002.pdf]

This article investigates the flexural fatigue behavior of FDM-3D printed PLA-wood composites. The following issues need to be addressed before publication:

1. The manuscript contains grammatical issues that need correction throughout the text..
2. Enhance the abstract by emphasizing key quantitative results and findings.
3. Given the absence of a pure PLA sample in the current study, exclude information related to pure PLA printing from Table 2.
4. Authors should explicitly state the equivalence of the mechanical properties of the PLA employed in the PLA-Wood filament from Shenzhen eSUN with the mechanical properties of PLA printed at the AMB laboratory. If the grades and properties match, a comparative analysis of their fatigue data can be conducted.
5. Revise the caption for Table 4 to clarify that it presents fatigue parameters resulting from regression analysis for all composite PLA-wood data, with no correlation to the fatigue parameters of PLA.
6. Referring to Figure 5, the results of the fatigue life to weight ratio for PLA-wood have been compared with all the results of fatigue life to weight ratio for PLA under various printing conditions. It is advisable to present the comparison of fatigue life to weight ratio between PLA-wood and PLA under consistent printing conditions in a tabular format. This will allow for a more accurate and quantitative comparison of these results under similar printing conditions.
7. Provide a more detailed analysis and interpretation of why 3D-printed PLA-wood samples exhibit superior fatigue life compared to PLA under various printing conditions.
8. To achieve a more accurate identification of different phases in the fractured cross-sectional images, consider conducting an EDS point analysis for precise characterization of the various phases.
