# Supplementary material for: Evaluation of stress-controlled high-cycle fatigue characteristics in PLA-wood fused deposition modeling 3D-printed parts under bending loads
Source: PLoS One. 2024 Apr 18;19(4):e0300569. doi: 10.1371/journal.pone.0300569 (PMC11025761; doi:10.1371/journal.pone.0300569)
Supplement: Attachment — Submitted filename: Respond to Comments.docx [file pone.0300569.s003.docx]

**Dear Editor-in-Chief,**

Journal of PLOS ONE,

Our manuscript, entitled "**Evaluation of stress-controlled high-cycle fatigue characteristics in PLA-wood fused deposition modeling 3D-printed parts under bending loads**," has undergone a thorough review by esteemed reviewers. Firstly, we express our gratitude to the reviewers for their valuable insights. The authors have diligently endeavored to incorporate all suggested revisions, clearly marking the modifications with yellow highlights. Additionally, detailed responses to each comment are presented in the subsequent paragraphs.

Regards,

**M. Azadi, Ph.D.**

Faculty of Mechanical Engineering, Semnan University, Semnan, Iran

**Reviewer #1:**

The authors study the evaluation of stress-controlled high-cycle fatigue characteristics in PLA-wood fused deposition modeling 3D-printed components under bending loads. The manuscript had an interesting topic and was well-written, however, it could only be accepted with the following corrections:

**Answer:** The authors extend their gratitude to the esteemed reviewer for providing valuable comments. We would like to confirm that all the comments raised have been thoroughly addressed and incorporated into the revised article.

1. For the title, it is suggested to change ‘3D-printed components’ to ‘3D-printed parts’. The word "components" might lead readers to get confused.

**Answer:** The mentioned term has been corrected as follows:

**Evaluation of stress-controlled high-cycle fatigue characteristics in PLA-wood fused deposition modeling 3D-printed parts under bending loads**

2. In abstract: a) ‘Therefore, Solid fatigue test’- ‘solid’ should be lower case. b) it is better to provide a scientific value or data of the result.

**Answer:** The ‘solid’ term has been edited and the following sentences are added in the revised article as follows:

The fatigue lifetime of the PLA-wood was decreased by 87.48% at the highest stress level (15 MPa) rather than the lowest stress level (7.5 MPa). Additionally, the results …

3. FFF (fused filament fabrication) can be mentioned, which is used on a par with FDM.

**Answer:** The FFF term is defined in the Introduction section as follows:

FDM or fused deposition modeling, which is also known as the fused filament fabrication (FFF), is a prevalent AM method …

4. The content of the paper looks similar to previous review papers. Does this paper same with previous studies? If not, please highlight the differences. What is ‘wow’ factors in this paper?

**Answer:** Considering the next comment (#5) of the respected reviewer, the authors are provided the following paragraph to cover both comments:

The literature review underscored the increasing utilization of PLA-woods and highlighted the significance of assessing their performance under cyclic loading. Nonetheless, there has been limited exploration into the rotary bending fatigue behavior of the aforementioned material, while the obtained results were unfavorable. However, in this study the proposed printing parameters and fatigue lifetime of the examined PLA-wood filament were found to be commendable, exhibiting comparability with PLA.

5. Research gap was not found in the text (last paragraph from the introduction).

**Answer:** The following paragraph is added to the revised article:

The literature review underscored the increasing utilization of PLA-woods and highlighted the significance of assessing their performance under cyclic loading. Nonetheless, there has been limited exploration into the rotary bending fatigue behavior of the aforementioned material, while the obtained results were unfavorable. However, in this study the proposed printing parameters and fatigue lifetime of the examined PLA-wood filament were found to be commendable, exhibiting comparability with PLA.

6. It is possible to cite the following papers in introduction section as references:

• Analysis on dimensional accuracy of 3D printed parts by Taguchi approach. Advances in Mechatronics, Manufacturing, and Mechanical Engineering. Lecture Notes in Mechanical Engineering, pp. 219-231 (2021), Springer Singapore.

• Rheological Properties of Natural Fiber Reinforced Thermoplastic Composite for Fused Deposition Modeling (FDM): A Short Review. Journal of Advanced Research in Fluid Mechanics and Thermal Sciences 98(2) (2022): 157-164.

**Answer:** The mentioned papers are cited in the Introduction section as follows:

Ahmad et al. [12] studied enhancing the performance of 3D printed bio-composite polymers, particularly focusing on natural fiber-based feedstock filaments for FDM. This paper emphasized the need for a rheological analysis of these new composite materials derived from natural resources. It aimed to characterize their flow behavior and the optimal melting temperature for the extrusion process in wire filament production. The primary focus of this study was on the rheological properties of fiber-reinforced thermoplastic composites intended for FDM. Moreover, Ahmad and Mohamad [13] optimized the accuracy of 3D-printed parts by applying the Taguchi method, considering variations induced by machine process parameters such as quality, temperature, and speed. Comparison of CMM results with CAD data, coupled with Taguchi analysis, identified honeycomb print pattern, 0 y-axes orientation, 90° support angle, and 0.4 sidewalk offset as the optimum parameters. The print pattern exhibited the most significant impact on part dimension accuracy, and the optimized parameters led to a 44.3% reduction in shrinkage, improving accuracy by 0.190 mm.

7. Highlight the model of 3D printer and specification.

**Answer:** The following sentences are added in the revised article:

The FDM 3D printer device was designed and built in the advanced material behavior (AMB) research laboratory (Semnan University, Semnan, Iran). It was developed with three axes: the extruder moves on the X-Y plane with a belt driving system, and the build platform moves on the Z axis with a lead screw driving system. It can print parts up to 550 × 600 × 700 mm³ with an accuracy of 50 μm on the X-Y axes and 40 μm on the Z axis. The printer uses a NEMA 17 stepper motor, a driver with adjustable micro step resolutions, and the Marlin firmware [31].

8. The authors used a commercial filament, PLA/wood. What is the composition of the wood?

**Answer:** Considering the respected reviewer’s comment, the percentage of wood filler is mentioned in the revised article:

The percentage of wood filler in the PLA matrix was 9% [29, 30].

9. Figure 4, why the number of samples between PLA and PLA/wood were unbalance or unequal?

**Answer:** For better understanding, the sentence ‘For more comparison, the S-N curve was compared with the former fatigue experiment results of pure PLA performed in the AMB Laboratory.’ is modified in the revised article as follows:

The fatigue lifetime of PLA-wood was compared to the fatigue behavior of pure PLA samples produced with various printing parameters used in previous research conducted at the AMB laboratory, emphasizing the excellent comparability of the PLA-wood printed samples.

10. The conclusion could be improved by adding the limitations and implications for researchers.

**Answer:** The following sentences are added to the conclusion section of the revised article:

- The $\sigma_{f}^{'}$ values for the studied samples were 294.60 and 353.04, based on all data and averaged values, respectively. Similarly, the $b$ values for the studied samples were -0.274 and -0.320, based on all data and averaged values, respectively.
- The PLA-wood exhibited a significant 87.48% decrease in fatigue lifetime when subjected to the highest stress level (15 MPa) compared to the lowest stress level (7.5 MPa).

11. The authors should update the reference list (latest references from the year of 2019-2024)

**Answer:** Considering the respected reviewer’s comment, the following references are cited in the revised article:

[12] Ahmad, M. N., Ishak, M. R., Taha, M. M., Mustapha, F., & Leman, Z. (2022). Rheological Properties of Natural Fiber Reinforced Thermoplastic Composite for Fused Deposition Modeling (FDM): A Short Review. *Journal of Advanced Research in Fluid Mechanics and Thermal Sciences*, *98*(2), 157-164.

[13] Ahmad, M. N., & Mohamad, A. R. (2021). Analysis on dimensional accuracy of 3D printed parts by Taguchi approach. In *Advances in Mechatronics, Manufacturing, and Mechanical Engineering: Selected articles from MUCET 2019* (pp. 219-231). Springer Singapore.

[30] Sultana, J., Rahman, M. M., Wang, Y., Ahmed, A., & Xiaohu, C. (2023). Influences of 3D printing parameters on the mechanical properties of wood PLA filament: an experimental analysis by Taguchi method. *Progress in Additive Manufacturing*, 1-13.

[31] Dadashi, A., & Azadi, M. (2024). Optimization of 3D printing parameters in polylactic acid bio-metamaterial under cyclic bending loading considering fracture features. *Heliyon*.

[33] Aghareb Parast, M. S., Jamalkhani Khameneh, M., Azadi, M., Azadi, M., Mahdipanah, M. H., & Roostaie, S. (2021). Effect of plasma nitriding on high‐cycle fatigue properties and fracture behaviors of GJS700 nodular cast iron under cyclic bending loading. *Fatigue & Fracture of Engineering Materials & Structures*, *44*(8), 2070-2086.

[50] Sachin, S. R., Kannan, T. K., & Rajasekar, R. (2020). Effect of wood particulate size on the mechanical properties of PLA biocomposite. *Pigment & Resin Technology*, *49*(6), 465-472.

[52] Kain, S., Ecker, J. V., Haider, A., Musso, M., & Petutschnigg, A. (2020). Effects of the infill pattern on mechanical properties of fused layer modeling (FLM) 3D printed wood/polylactic acid (PLA) composites. *European journal of wood and wood products*, *78*, 65-74.

[53] Kuciel, S., Mazur, K., & Hebda, M. (2020). The influence of wood and basalt fibres on mechanical, thermal and hydrothermal properties of PLA composites. *Journal of Polymers and the Environment*, *28*, 1204-1215.

[55] Bagheri, A., Parast, M. S. A., Kami, A., Azadi, M., & Asghari, V. (2022). Fatigue testing on rotary friction-welded joints between solid ABS and 3D-printed PLA and ABS. *European Journal of Mechanics-A/Solids*, *96*, 104713.

[63] Azadi, M., Aroo, H., & Parast, M. S. A. (2021). Comparing of High-Cycle Fatigue Lifetimes in Un-corroded and Corroded Piston Aluminum Alloys in Diesel Engine Applications. *Archives of Foundry Engineering*, *21*.

12. There were a few typos in the text, which should be paid attention to (please proofread).

**Answer:** The revised manuscript is revised and rechecked with the Grammarly software and its score is 98.

**Reviewer #2:**

This article investigates the flexural fatigue behavior of FDM-3D printed PLA-wood composites. The following issues need to be addressed before publication:

**Answer:** The authors extend their gratitude to the esteemed reviewer for providing valuable comments. We would like to confirm that all the comments raised have been thoroughly addressed and incorporated into the revised article.

1. The manuscript contains grammatical issues that need correction throughout the text.

**Answer:** The revised manuscript is rechecked with Grammarly and its score is 98.

2. Enhance the abstract by emphasizing key quantitative results and findings.

**Answer:** The following sentences are added in the revised article as follows:

The fatigue lifetime of the PLA-wood was decreased by 87.48% at the highest stress level (15 MPa) rather than the lowest stress level (7.5 MPa). Additionally, the results …

3. Given the absence of a 3D-printed pure PLA sample in the current study, exclude information related to pure PLA 3D printing material from Table 2.

**Answer:** Considering the respected reviewer’s comment, Table 2 is modified in the revised article:

Table 2. 3D-printing parameters of PLA-wood samples fabricated with the FDM method

| Ref. | Material | Print parameters | | | | | | | |
| --- | --- | --- | --- | --- | --- | --- | --- | --- | --- |
|  |  | Nozzle Temp. ($℃$) | Bed Temp. ($℃$) | Nozzle Diameter (mm) | Layer Height (mm) | Infill (%) | Infill Pattern | Print Speed (mm/s) | Print Direction |
| [35] | PLA-wood | 240 | 90 | 0.4 | 0.2 | 100 | - | - | H |
| [36] |  | 200 | 60 | 0.4, 0.6 | 0.15 | 100 | - | 50 | - |
| [37] |  | 200 | 80 | 0.4 | 0.05, 0.1, 0.2, 0.3 | 100 | - | - | H |
| [38] |  | 180, 200, 220 | - | 0.4 | 0.1, 0.3 | 100 | - | 30, 40, 50 | H |
| [27] |  | 180 | 50 | 0.5, 0.6, 0.7 | 0.2, 0.3, 0.4 | 25, 50, 75 | HC., Rect. | 25, 30, 35 | - |
| [39] |  | 210 | 60 | - | 0.3 | 80 | HC. | 3 | - |
| [40] |  | - | - | 0.8 | 0.2 | - | - | 30 | - |

*Abbreviation: "Rect" means Rectangular, "HC" means Honeycomb, and "H" means Horizontal.

4. Authors should explicitly state the equivalence of the mechanical properties of the PLA employed in the PLA-Wood filament from Shenzhen eSUN with the mechanical properties of PLA printed at the AMB laboratory. If the grades and properties match, a comparative analysis of their fatigue data can be conducted.

**Answer:** To answer this comment, the following sentences and Table 3 are added to the revised article:

Moreover, the mechanical properties of PLA filament used in AMB laboratory (YouSu Company), and PLA filament produced by eSUN Company were compared in Table 3.

Table 3. The mechanical properties of PLA filament produced by YouSu Company and eSUN Company

| Mechanical property | YouSu Company [45] | eSUN Company [46] |
| --- | --- | --- |
| Density (g/cm^3^) | 1.24 | 1.24 |
| Melt flow index (g/10min) | 7 (210$℃$/2.16kg) | 5.2 (190$℃$/2.16 kg) |
| Tensile Strength (MPa) | 60 | 60 |
| IZOD Impact Strength | 16 (J/m) | 4.3 (kJ/m^2^) |

5. Revise the caption for Table 4 to clarify that it presents fatigue parameters resulting from regression analysis for all composite PLA-wood data, with no correlation to the fatigue parameters of PLA.

**Answer:** The mentioned point is corrected as follows:

Table 5. The fatigue properties of PLA-wood according to all experimental data and averaged values

| All Data | | | Averaged values | | |
| --- | --- | --- | --- | --- | --- |
| $\sigma_{f}^{'}$ [MPa] | *b* [-] | R^2^ [%] | $\sigma_{f}^{'}$ [MPa] | *b* [-] | R^2^ [%] |
| 294.60 | -0.274 | 0.828 | 353.04 | -0.320 | 0.989 |

6. Referring to Figure 5, the results of the fatigue life to weight ratio for PLA-wood have been compared with all the results of fatigue life to weight ratio for PLA under various printing conditions. It is advisable to present the comparison of fatigue life to weight ratio between PLA-wood and PLA under consistent printing conditions in a tabular format. This will allow for a more accurate and quantitative comparison of these results under similar printing conditions.

**Answer:** Due to the unavailability of precise printed parameters for both PLA and PLA wood, the PLA wood data was compared against a range of printed PLA samples. This is the reason for utilizing the S-N curve in the analysis.

7. Provide a more detailed analysis and interpretation of why 3D-printed PLA-wood samples exhibit superior fatigue life compared to PLA under various printing conditions.

**Answer:** To answer this comment, the following sentences are added to the revised article:

The addition of wood to PLA can have various effects on its fatigue properties. Firstly, the inclusion of wood fibers often increases the overall stiffness of the PLA matrix. This leads to a composite material with improved rigidity compared to pure PLA. The increased stiffness can help the material resist deformation and fatigue failure under cyclic loading [48, 49].

Additionally, the presence of wood particles can influence the tensile strength of the composite. While the exact effect can depend on factors such as the size, shape, and distribution of the wood particles [50], in many cases, the tensile strength of the composite can be enhanced compared to pure PLA [23, 51]. For instance, Kain et al. [52] showed that the higher the fiber content in the filament can lead to better mechanical properties. Moreover, Kuciel et al. [53] studied the impact of wood and basalt fibers on the mechanical and hydrothermal properties of PLA composites and indicated the Young modulus of composites improved by 45% with the addition of 15 wt% Woof Fibers.

However, it's important to note that the incorporation of wood particles can also introduce directional dependencies in the mechanical characteristics of PLA. This means that the material's properties may vary depending on the direction in which it is tested. These directional dependencies can potentially affect the material's fatigue behavior, as the distribution of stress and strain within the material can be influenced by the orientation of the wood particles. Overall, the addition of wood to PLA can result in a composite material with improved stiffness, tensile strength, and potentially complex fatigue behavior due to the presence of directional dependencies.

8. To achieve a more accurate identification of different phases in the fractured cross-sectional images, consider conducting an EDS point analysis for precise characterization of the various phases.

**Answer:** Considering the respected reviewer’s comment, Figure 10 is added to the revised article:

For precise characterization of the various phases, the EDS point analysis of the fracture surface of PLA-wood was shown in Figure 10.

Figure 10. EDS point analysis of fracture surface of PLA-wood
